# Enteropathogenic Potential of *Bacillus thuringiensis* Isolates from Soil, Animals, Food and Biopesticides

**DOI:** 10.3390/foods9101484

**Published:** 2020-10-17

**Authors:** Valerie Schwenk, Janina Riegg, Monique Lacroix, Erwin Märtlbauer, Nadja Jessberger

**Affiliations:** 1 Department of Veterinary Sciences, Faculty of Veterinary Medicine, Ludwig-Maximilians-Universität München, Schönleutnerstr. 8, 85764 Oberschleißheim, Germany; Valerie.Schwenk@mh.vetmed.uni-muenchen.de (V.S.); janina.riegg@gmx.net (J.R.); e.maertlbauer@mh.vetmed.uni-muenchen.de (E.M.); 2 Centre Armand-Frappier Santé Biotechnologie, 531, boul. des Prairies, Laval, QC H7V 1B7, Canada; Monique.Lacroix@iaf.inrs.ca

**Keywords:** *Bacillus thuringiensis*, *Bacillus cereus*, food infections, enterotoxins, cytotoxicity, essential oils, risk evaluation, biopesticide

## Abstract

Despite its benefits as biological insecticide, *Bacillus thuringiensis* bears enterotoxins, which can be responsible for a diarrhoeal type of food poisoning. Thus, all 24 isolates from foodstuffs, animals, soil and commercially used biopesticides tested in this study showed the genetic prerequisites necessary to provoke the disease. Moreover, though highly strain-specific, various isolates were able to germinate and also to actively move, which are further requirements for the onset of the disease. Most importantly, all isolates could grow under simulated intestinal conditions and produce significant amounts of enterotoxins. Cytotoxicity assays classified 14 isolates as highly, eight as medium and only two as low toxic. Additionally, growth inhibition by essential oils (EOs) was investigated as preventive measure against putatively enteropathogenic *B. thuringiensis*. Cinnamon Chinese cassia showed the highest antimicrobial activity, followed by citral, oregano and winter savory. In all tests, high strain-specific variations appeared and must be taken into account when evaluating the hazardous potential of *B. thuringiensis* and using EOs as antimicrobials. Altogether, the present study shows a non-negligible pathogenic potential of *B. thuringiensis*, independently from the origin of isolation. Generally, biopesticide strains were indistinguishable from other isolates. Thus, the use of these pesticides might indeed increase the risk for consumers’ health. Until complete information about the safety of the applied strains and formulations is available, consumers or manufacturers might benefit from the antimicrobial activity of EOs to reduce the level of contamination.

## 1. Introduction

*Bacillus thuringiensis* is a Gram-positive and spore-forming bacterium, which is used worldwide as biopesticide due to its capability to produce insecticidal proteins [1,2,3]. The two main parasporal toxins are Cyt (cytolytic) and Cry (crystal) toxins [4]. In addition to that, *B. thuringiensis* produces other entomopathogenic factors such as vegetative insecticidal proteins (Vip) and secreted insecticidal proteins (Sip) [1,5,6]. As a result of its distinct types of toxins, *B. thuringiensis* is virulent to a wide range of insect orders and species, e.g., *Coleoptera*, *Diptera* and *Lepidoptera*, as well as nematodes and even plant and animal pathogenic microorganisms [2,7,8]. A clear advantage is the host-specific mode of action of the mentioned toxins, which do rarely harm non-target species.

Despite the benefits, the close relation to other members of the *Bacillus cereus* group must not be neglected. The *B. cereus* group includes at least eight species: *B. cereus sensu stricto*, *B. anthracis*, *B. weihenstephanensis*, *B. mycoides*, *B. pseudomycoides*, *B. cytotoxicus*, *B. toyonensis* and *B. thuringiensis* [9,10,11,12], while recently, more and more additional species are described. *B. cereus s. s.* is a human pathogen that causes two types of foodborne diseases, the emetic syndrome due to cereulide intoxication and the diarrhoeal type of disease [12]. The latter arises from an infection with vegetative cells, which produce enterotoxins, mainly the non-haemolytic enterotoxin (Nhe), haemolysin BL (Hbl) and cytotoxin K (CytK), in the human intestine [13,14,15,16,17]. Like *B. cereus*, *B. thuringiensis* exhibits the enterotoxin genes *nhe, hbl* and *cytK* [18,19,20]. Moreover, *B. thuringiensis* could be related to food-borne outbreaks [21]. These facts led to the debate if the biopesticides represent a risk for public health and thus, it seems questionable using potential harmful preparations extensively in agriculture. A further problem is that routine clinical and food diagnostics does not usually differentiate the *B. cereus* group species [22,23]. Thus, food-borne diseases and clinical infections caused by *B. thuringiensis* might be significantly under-reported. The aim of this study was to investigate the toxic and enteropathogenic potential of *B. thuringiensis* isolates gained from food, animal and soil samples, as well as from commercially used biopesticides. These 24 isolates were systematically characterized regarding their phylogenetic relationship, occurrence of toxin genes, growth and enterotoxin production under simulated intestinal conditions, cytotoxic activity, as well as further pathogenicity factors involved in the onset of *B. cereus*-associated food infections, such as spore germination and motility. Moreover, growth inhibition by essential oils (EOs) was investigated as preventive measure against putatively enteropathogenic *B. thuringiensis*.

## 2. Materials and Methods

### 2.1. Bacterial Strains and Culture Conditions

The *B. thuringiensis* strains used in this study are listed in Table 1. For gaining cell-free culture supernatants, they were pre-cultured in casein glucose yeast (CGY) medium with 1% glucose for 17 h at 32 °C and 200 rpm. Samples of 20 mL fresh CGY medium were inoculated to an optical density (OD_600_) = 0.2 and cultured for further 6 h. After centrifugation at 3500 rpm and 4 °C for 12 min, 1 mM ethylenediaminetetraacetic acid (EDTA) was added, the supernatants were filtered through a 0.2 µm filter, aliquoted and stored at −20 °C. For cultivation under simulated intestinal conditions, preparation of “conditioned” (cRPMI) medium was carried out as described previously [15]. In brief, RPMI 1640 medium (with stable glutamine; Biochrom AG, Berlin, Germany), supplemented with 1% glucose and 2% casein hydrolysate, was incubated with differentiated CaCo-2 cells for 22 h and filtered using a 0.2 µm filter. Overnight cultures obtained as described above were incubated at 37 °C and adjusted to a start OD_600_ of 0.05 in 20 mL cRPMI. They were incubated statically at 37 °C and 7% CO_2_ atmosphere. For growth analyses, OD_600_ was measured every hour for eight hours. Cell-free culture supernatants were harvested after six hours as described above.

### 2.2. Cell Lines and Culture Conditions

CaCo-2 cells from DSMZ (German Collection of Microorganisms and Cell Cultures, Braunschweig, Lower Saxony, Germany) were cultivated in RPMI 1640 medium (with stable glutamine) supplemented with 10% foetal bovine serum (Biochrom AG, Berlin, Germany). For cultivation, 80 cm^2^ culture flasks and a humidified incubator (37 °C and 7% CO_2_) were used. Cells were passaged 1:6 every three to four days. For differentiation, cells were incubated for 14 days. Medium was changed every three to four days.

### 2.3. PCR Analyses

Generally, amplification was performed with a total volume of 50 µL in a Biometra thermocylcer (Analytik Jena, Jena, Thuringia, Germany). The reaction mixtures contained 5 µL 10× buffer, 4 µL MgCl_2_ (25 mM), 1 µL dNTP suspension (10 mM each), 0.5 µL Taq DNA polymerase, 2.5 µL of each primer (10 µM) and 2 µL template DNA (colony material). Initial denaturation at 95 °C for five minutes was followed by 30 reaction cycles: denaturation at 95 °C for 15–60 s, annealing for 15–60 s at the appropriate temperature (see Table S1) and extension for 20–60 s at 72 °C. The final extension was performed at 72 °C for seven minutes. PCR fragments were separated on 1% agarose gels by electrophoresis and made visible by UV illumination. Primers used for all PCR analyses are listed in Table S1. First, the *panC* (pantothenate synthetase) gene was amplified for sequence analysis (see Section 2.4). Subsequently, all strains were screened for *cry* toxin genes and allocated to five groups according to Ben-Dov et al. [24]. Furthermore, the strain set was tested for the presence of the (entero)toxin genes *hblC*, *nheA*, *ces* and *cytK1* via multiplex PCR according to Wehrle et al. [25], as well as *cytK2* according to Ehling-Schulz et al. [26].

### 2.4. PanC Sequence Typing

To allocate the *B. thuringiensis* isolates tested in this study to phylogenetic groups, sequencing of a 485 bp fragment of the pantothenate synthetase gene (*panC*; [27]) was applied (Eurofins Genomics Germany GmbH, Ebersberg, Bavaria, Germany). Sequences were aligned using CLUSTAL Ω [28]. Further cluster analysis was carried out using SplitsTree software (version 4, Eberhard Karls University Tübingen, Tübingen, Baden-Württemberg, Germany ) [29]. The sequences of the following reference strains were added to the analysis: type I: *B. pseudomycoides* 219,298 (CP007626.1); type II: *B. wiedmannii* SR52 (CP032365.1); type III: *B. anthracis* Ames (AE016879.1); type IV: *B. cereus* ATCC 14579 (NC_004722.1); type V: *B. toyonensis* BCT-7112 (CP006863.1); type VI: *B. weihenstephanensis* WSBC 10,204 (DQ301426.1); type VII: *B. cytotoxicus* NVH391-98 (CP000764.1) [23,27,30].

### 2.5. Spore Preparation

*B. thuringiensis* spores were prepared as described previously [14,31,32]. Briefly, 100 mL of minimal sporulation medium (1 mM MgCl_2_ × 6 H_2_O, 1 mM Ca(NO_3_)_2_ × 4 H_2_O, 12.5 µM ZnCl_2_, 2.5 µM CuCl_2_, 2.5 µM CoCl_2_ × 6 H_2_O, 2.5 µM Na_2_MoO_4_ × 2 H_2_O, 5 µM (NH_4_)_2_SO_4_, 0.066 µM MnSO_4_ × H_2_O, 1 mM FeSO_4_ and 1 M maltose in bacto nutrient broth (Oxoid, Wesel, Northrhine-Westphalia, Germany); pH 7.6) were inoculated with 0.5% overnight culture (see Section 2.1) and incubated for three days at 32 °C and 200 rpm. Sporulation was monitored via bright-field microscopy. When >80% spore content was reached, the cultures were centrifuged at 3500 rpm and 4 °C for 10 min, washed three times in pre-cooled spore washing buffer (1 M KH_2_PO_4_, 1 M K_2_HPO_4_, pH 7.4) and stored at 4 °C.

### 2.6. Germination

For assessing germination of *B. thuringiensis* spores, media were inoculated to OD_600_ = 1. Two biological with three technical replicates for each strain were incubated in 96-well plates at 37 °C for one hour. As described before [14], OD_620_ was measured every three minutes in a Tecan photometer using Ridawin software. Germination is shown as the decrease of OD_620_ (in %) per time. Germination was compared in CGY and cRPMI medium, as well as with and without prior heat activation for 10 min at 80 °C.

### 2.7. Motility Assay

To evaluate swimming behaviour of the *B. thuringiensis* strains, three replicates each were tested. For this, 1 µL CGY overnight culture was injected into the centre of 53 mm diameter CGY agar plates containing 0.25% agar and incubated at 37 °C. Motility diameters were assessed after 24 h.

### 2.8. Enzyme Immunoassays (EIAs)

Sandwich enzyme immunoassays for the detection of the enterotoxin components NheB and Hbl L2 were performed as described previously [33,34,35]. The following antibodies were used for detection: 5 µg/mL mAb 2B11/1E11-HRP 1:2000 (NheB) and 10 µg/mL mAb 1A12/1H9-HRP 1:1000 (Hbl L2). Titres are defined as the reciprocal of the highest dilutions resulting in an absorbance value of ≥1.0. Results were compared according to those titres, which were calculated from at least three replicates each.

### 2.9. WST-1 Bioassay

WST-1 bioassays were performed on CaCo-2 cells as described earlier [15]. Briefly, serial dilutions of the culture supernatants were applied to 96-well plates in RPMI 1640 medium (100 µL/well) before 2 × 10^4^ three days old CaCo-2 cells/well (100 µL) were added. After 24 h incubation at 37 °C and 7% CO_2_, cell viability was determined by addition of the cell proliferation regent WST-1 (water soluble tetrazolium, Roche diagnostics, Penzberg, Bavaria, Germany). Increasing amounts of formazan were detected in a Tecan infinite F50 photometer at 450 nm. Dose-response curves and thus, 50% lethal concentrations of at least two replicates were calculated with Magellan software and are shown as reciprocal titres.

### 2.10. Haemolysis Assays

CGY culture supernatants (10 µL) were filled into stamp holes with five mm diameter on sheep blood agar plates. After five hours incubation at 32 °C, haemolysis patterns were assessed.

### 2.11. Disk Diffusion Assays

To investigate antimicrobial effects of essential oils (EOs) on the *B. thuringiensis* isolates, disk diffusion assays were carried out according to Dussault et al. [36]. Cinnamon Chinese cassia and cinnamon OR (*Cinnamomum cassia*), citral (pure; 2,6-octadienal,3,7-dimethyl), oregano (*Thymus capitatus* Hoff.) and thyme (red; *Thymus vulgaris* and *Thymus zygis* L. var. *gacilis* Boissier) were provided by BSA Food Ingredients s.e.c./l.p. (Montréal, Canada). Winter savory (*Satureja montana* L.) was purchased from Alixir Essential Oils (Grondines, Canada). For the disk diffusion assays, CGY overnight cultures (see Section 2.1) were set to OD_600_ = 0.5 (approx. 10^7^ CFU/mL) and 100 µL were plated onto CGY agar. A sterile Whatmann paper disk (Ø 5 mm) was placed in the middle of the plate and 1 µL EO was dripped on it. After 24 h incubation at 37 °C, growth inhibition zones were measured. At least three replicates were prepared and examined.

### 2.12. Antimicrobial Broth Dilution Assays

EOs with antibacterial effects in the disk diffusion tests (see Section 2.11) were applied in antimicrobial broth dilution assays to determine minimal inhibitory as well as maximal tolerated concentrations according to Dussault et al. [36]. The EOs were set to a concentration of 2.5% (dissolved in sterile H_2_O with 5% Tween 80). The first column of a 96-well microtitre plates was filled with 167.7 µL CGY medium and 33.3 µL of the prepared EO solution. Columns two to 12 were filled with 100 µL CGY and a two-fold dilution series was prepared. EO concentrations thus ranged from 0.42 to 0.00041%. Column 12 was not filled with EOs and served as positive control. The *B. thuringiensis* CGY overnight cultures were set to OD_600_ = 0.5 (approx. 10^7^ CFU/mL) and further diluted to approx. 10^4^ CFU/mL. Rows A and H served as negative control, the remaining wells were filled with 10 µL bacterial suspension. For the determination of the minimal inhibitory concentration (MIC) a total of 10^6^ CFU/mL were used, 10^3^ CFU/mL to establish the maximal tolerated concentration (MTC). After 24 h incubation at 37 °C, 10 µL iodonitrotetrazolium chloride (Sigma-Aldrich/Merck KGaA, Darmstadt, Hesse, Germany) were added to each well. After further 60 min incubation at 37 °C, bacterial growth was measured at OD_540_ in a Tecan infinite F50 photometer. The MIC was calculated from the mean values of the lowest EO concentrations that prevented bacterial growth. The MTC was determined by the mean values of the maximal concentrations that allowed bacterial growth. The experiment was done twice with three technical replicates per strain.

### 2.13. Statistical Analyses

Data were statistically validated using the column statistics program of GraphPad Prism version 5.00 for Windows (GraphPad Software, San Diego, CA USA). Unpaired *t*-tests were applied with two-tailed *p*-values and 95% confidence interval. Significant differences were marked with * (*p*-value ≤ 0.05). Furthermore, Spearman correlation tests were performed with two-tailed *p*-values and 95% confidence interval to determine correlations between data sets. Significant correlation was marked with * (*p*-value ≤ 0.05).

## 3. Results

### 3.1. Genetic Prerequisites of the Tested Strains

**Figure 1:**
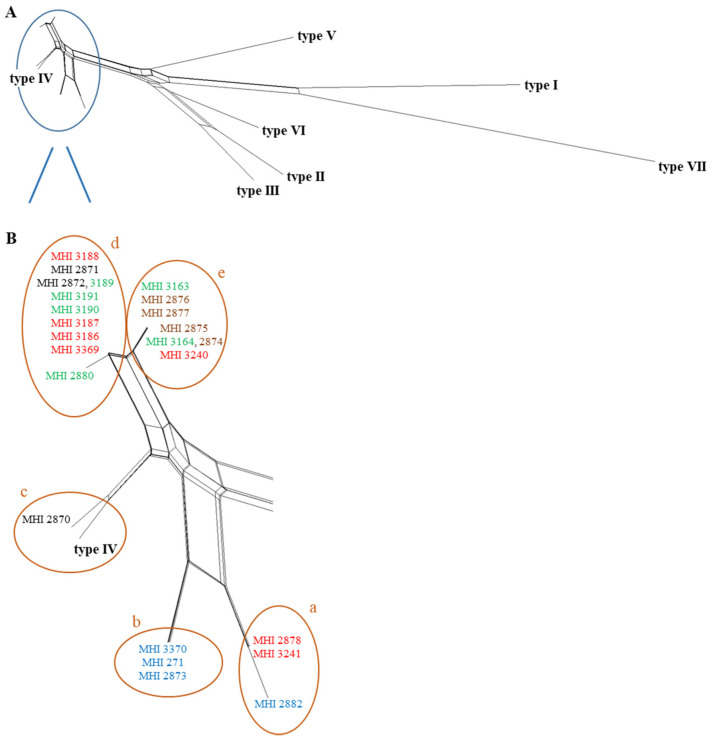
Phylogenetic relationship between the 24 tested *B. thuringiensis* strains, based on *panC* sequence analysis. Sequences were aligned with Clustal Ω [28] and further analysed using SplitsTree4 [29]. (**A**) Overview. (**B**) Enlargement of the *B. thuringiensis* isolates, which were all assigned to *panC* type IV. Within these, five distinct clusters (a–e) were identified. Blue: animal isolate, green: food, red: biopesticide, brown: soil, black: unknown origin.

In this study, 24 *B. thuringiensis* isolates of different origin (see Table 1) were characterized. For a rough overview on their phylogenetic relationship, the isolates were subjected to *panC* sequence typing, allocating them to *panC* type IV (Figure 1A). Distinct clusters within this group appeared (Figure 1B). In cluster a, MHI 2882 (*B. thuringiensis* ssp. *israelensis* isolated from larva) was found, as well as MHI 3241 and the older, barely documented isolate MHI 2878 (both *B. thuringiensis* ssp. *tenebrionis*), which might be identical. Cluster b comprised MHI 271, MHI 2873 (ATCC type strain) and MHI 3370. The reference strain *B. cereus* ATCC 14579 and MHI 2870 (*B. thuringiensis* ssp. *entomocidus*) belonged to cluster c. All further isolates clustered closer together. Cluster d comprised four biopesticidal strains and four food isolates, while in cluster e mainly soil and salad isolates appeared, with the exception of biopesticidal isolate MHI 3240. Interestingly, MHI 2872 and MHI 3189 (from ready-to-eat salad) in cluster d as well as MHI 2874 (*B. thuringiensis* ssp. *kurstaki*, isolated from soil) and MHI 3164 (also from ready-to-eat salad) in cluster e seemed to be identical.

Furthermore, all isolates were tested for the presence of *cry* genes via PCR with universal primers according to Ben-Dov et al. [24]. Thus, they could be verified as *B. thuringiensis* and additionally grouped in *cry*-type gene profiles (see Table 1). Twenty strains (83%) were classified as *cry1*, while 13 of those (54%) were also positive for *cry2*. Two strains (MHI 2878 and MHI 3241; *B. thuringiensis* ssp. *tenebrionis*) were allocated to the *cry3*-type and one strain (MHI 2882; Culicidae larva isolate) to *cry4*. MHI 3370, the ancrystalliferous derivate Bt 407 Cry^−^ [37,38], was negative for all *cry*-types. Equally, all isolates were screened for the presence of (entero)toxin genes. All isolates harboured *nheA* as well as *hblC*, but were negative for *ces* as well as *cytK1* (see Table 1). Twenty two strains (92%) were tested positive for *cytK2* and could thus be assigned to toxin profile A (*nhe*+, *hbl*+, *cytK2*+), while only two isolates (MHI 2878 and MHI 3241; *B. thuringiensis* ssp. *tenebrionis*) were *cytK2* negative and thus, allocated to toxin profile C (*nhe*+, *hbl*+) [39]. Interestingly, these were the two isolates assigned to the *cry3*-type. However, investigating a larger set of strains would be necessary to contextualize these observations.

### 3.2. Strain-Specific Germination and Motility

So far, all *B. thuringiensis* isolates in this study showed the genetic premises (presence of enterotoxin genes) to provoke the diarrhoeal type of food poisoning. Nevertheless, prerequisites for *B. cereus* enterotoxin production and cytotoxicity are also spore germination [14,40,41,42,43] and motility [14,44,45,46] in the host’s intestine.

**Figure 2:**
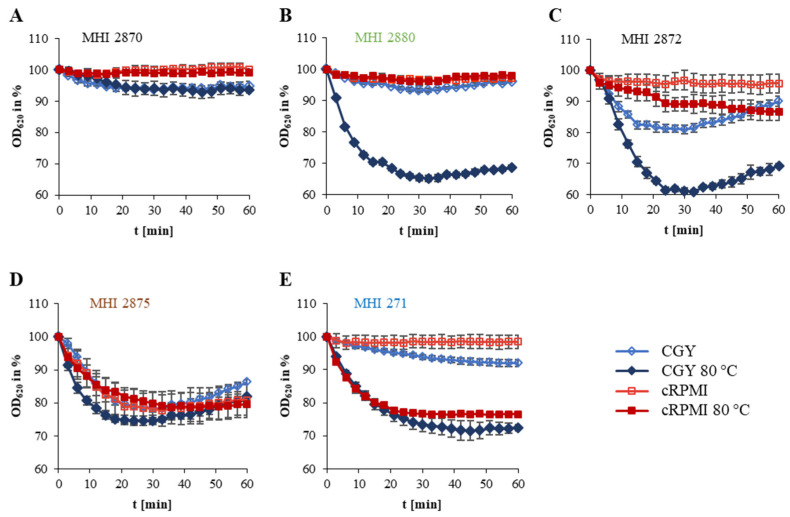
Germination of *B. thuringiensis* spores. Germination is depicted as the decrease of OD_620_ per unit of time. OD_620_ at time point 0 was set to 100%. Due to different patterns, strains were assigned to five germination types. (**A**) Minimal to no germination. (**B**) Strong germination in nutrient-rich medium plus heat activation with little germination in cRPMI medium. (**C**) Heat- and nutrient-dependent germination. (**D**) Heat- and nutrient-independent germination. (**E)** Germination heat-dependent, but mostly nutrient-independent. Blue: animal isolate, green: food, red: biopesticide, brown: soil, black: unknown origin. CGY: casein-glucose-yeast full medium. CGY 80 °C: casein-glucose-yeast full medium with prior heat activation for 10 min at 80 °C. cRPMI: RPMI 1640 cell culture medium pre-incubated with CaCo-2 cells. cRPMI 80 °C: RPMI 1640 cell culture medium pre-incubated with CaCo-2 cells and additional prior heat activation for 10 min at 80 °C.

Within the *B. thuringiensis* strain set a high variability was detected, from maximal germination of 45% in CGY medium with heat activation to no germination. Furthermore, different germination patterns emerged (see Figure 2 and Table 2). Pattern A is characterized by minimal or no germination (MHI 2870 and MHI 2873). Strong germination in nutrient-rich medium with heat activation, but little germination in cRPMI medium was classified as pattern B (six food isolates, three biopesticidal strains and MHI 2882 isolated from larva). Pattern C germination seemed to be heat- as well as nutrient-dependent. This was detected for spores of biopesticidal strains as well as isolates of unknown origin. Pattern D comprised mostly soil isolates, which showed heat- and nutrient-independent germination. Spores with heat-dependent, but mostly nutrient-independent germination were summarized in pattern E.

**Figure 3:**
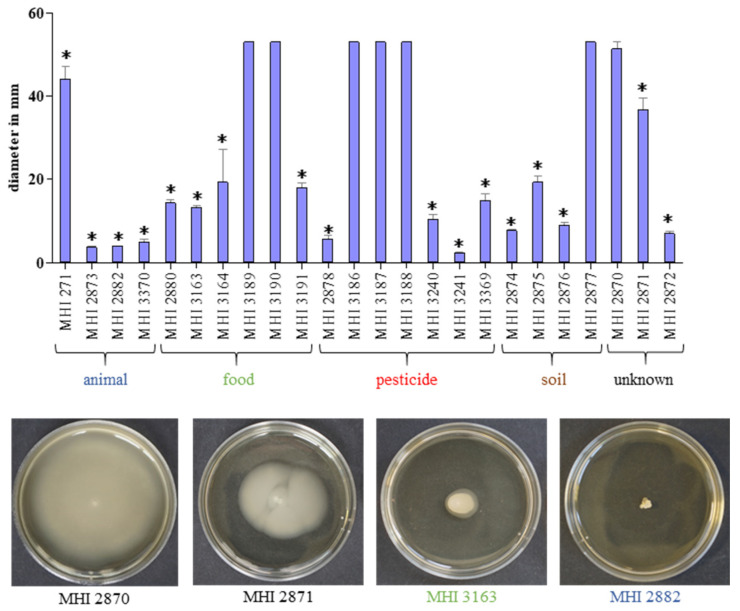
Motility of the *B. thuringiensis* isolates. The 1 μL overnight culture was injected into the centre of CGY plates with 0.25% agar. Swimming diameters were measured after 24 h incubation at 37 °C. *: Significant difference compared to full-plate expansion (*p*-value ≤ 0.05; unpaired *t*-test, 95% confidence interval). Blue: animal isolate, green: food, red: biopesticide, brown: soil, black: unknown origin.

Beyond germination, the swimming ability of the isolates was investigated using CGY plates with 0.25% agar. With the exception of insect isolates MHI 2873 and MHI 2882, and biopesticide isolate MHI 3241, all strains were able to actively move after 24 h incubation at 37 °C, while MHI 2870, as well as the salad and biopesticide isolates MHI 3186, MHI 3187, MHI 3188, MHI 3189 and MHI 3190 showed maximal diameters (Figure 3). Generally, various strains—including isolates from biopesticides—were able to germinate in low-nutrient cRMPI medium and also to actively move; hence, further prerequisites for the onset of the disease are given.

### 3.3. Growth under Simulated Intestinal Conditions and Production of Enterotoxins

**Figure 4:**
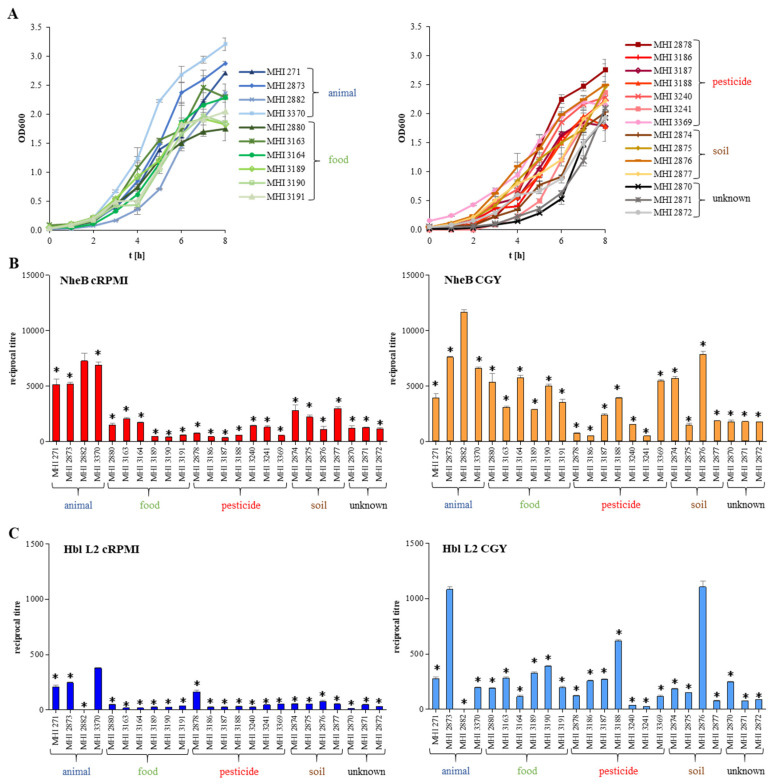
Growth and enterotoxin production of the *B. thuringiensis* strains. (**A**) Growth under simulated intestinal conditions (RPMI 1640 medium treated with CaCo-2 cells, 37 °C, 7% CO_2_). (**B**) NheB production after six hours growth under simulated intestinal conditions (cRPMI, red). Reciprocal NheB titres after six hours growth in CGY medium (nutrient-rich, aerobic conditions) are shown for comparison (orange). (**C**) Hbl L2 production after six hours growth under simulated intestinal conditions (cRPMI, dark blue). Reciprocal Hbl L2 titres after six hours growth in CGY medium (nutrient-rich, aerobic conditions) are shown for comparison (light blue). *: Significant difference compared to the isolate with highest toxin production (*p*-value ≤ 0.05; unpaired *t*-test, 95% confidence interval). Blue: animal isolate, green: food, red: biopesticide, brown: soil, black: unknown origin.

The crucial step for *B. cereus*-associated food infections is growth and enterotoxin production of the bacteria in the human intestine. Representatively, reciprocal titres of toxin components NheB and Hbl L2 were determined in culture supernatants after six hours of growth. All *B. thuringiensis* isolates tested in this study were able to grow under simulated intestinal conditions (cRPMI medium, 37 °C, 7% CO_2_) and to secrete significant amounts of NheB (Figure 4A,B). Applying a classification scheme developed for *B. cereus* (high NheB production: titres >2000; medium: 1000–2000; low: <1000; [15]), a uniform distribution of high, medium and low toxin producing strains was observed (see Table 1 and Figure 4B). Under these terms, eight strains (mainly biopesticide and salad isolates) showed NheB titres below 1000 (Figure 4B), but conclusions should be drawn carefully, as this is still sufficient to cause significant damage to CaCo-2 cells (see Section 3.4). Additionally, NheB titres were determined after six hours growth in CGY medium, as this is applied in routine diagnostics of *B. cereus* [39]. Due to accelerated growth, titres were enhanced in CGY medium compared to cRPMI. Furthermore, all isolates secreted significant amounts of Hbl L2 under both conditions, similar to *B. cereus* [15,39], with the exception of Culicidae larva isolate MHI 2882 (Figure 4C).

### 3.4. Exhibition of Cytotoxic and Haemolytic Activity

**Figure 5:**
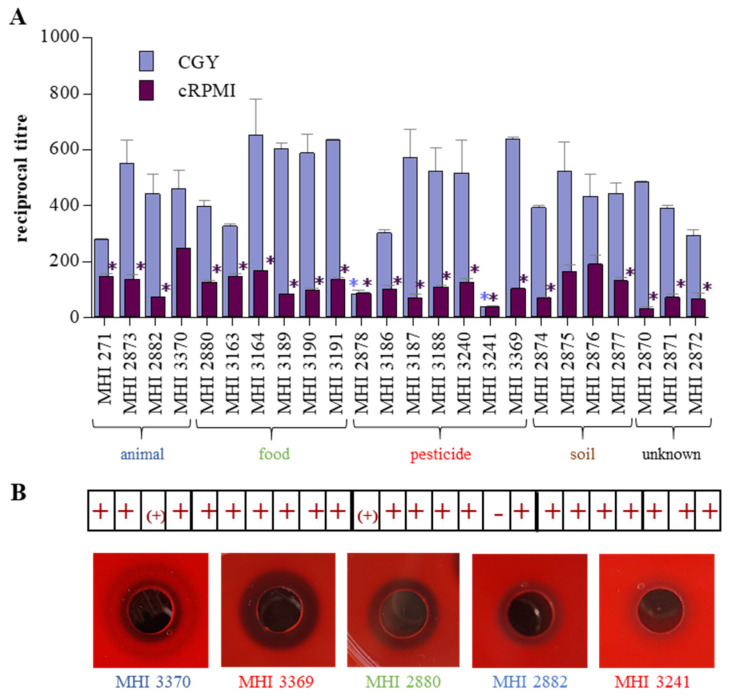
Cytotoxic activity of the 24 tested *B. thuringiensis* isolates. **A.** Results of WST-1 bioassays on CaCo-2 cells. Reciprocal titres for 50% lethal doses are shown. *: Significant difference compared to the isolate with highest cytotoxicity (*p*-value ≤ 0.05; unpaired *t*-test, 95% confidence interval). **B.** Haemolytic activity of the 24 isolates obtained after five hours incubation on sheep blood agar plates. +: Haemolysis clearly detectable. −: Haemolysis not detectable. (+): Weak haemolysis. Further, examples of five different haemolysis patterns are shown. Blue: animal isolate, green: food, red: biopesticide, brown: soil, black: unknown origin.

Beyond toxin production, all tested *B. thuringiensis* isolates grown under simulated intestinal conditions showed cytotoxic activity towards the human colon adenocarcinoma cell line CaCo-2 (Figure 5A). According to a prior classification of *B. cereus* strains [15], 14 of the tested *B. thuringiensis* isolates were classified as highly (titres > 100) and eight isolates as medium (titres from 50 to 100) toxic (see Table 1). Only two strains exhibited low cytotoxicity (titres < 50), which were MHI 2870 and biopesticide isolate MHI 3241. Similar to toxin production (see Section 3.3), cytotoxicity titres where accordingly enhanced when the strains were grown in CGY medium (Figure 5A). Data from WST-1 bioassays were supported by haemolysis (Figure 5B). Only strain MHI 3241 exhibited no haemolytic activity on sheep blood agar. Extension of the haemolytic zone varied strain-specifically, with isolates MHI 271, MHI 2873 and MHI 3370 showing the discontinuous haemolysis zone phenomenon [13,47,48]. Furthermore, data obtained from EIAs and WST-1 bioassays were statistically analysed. Spearman correlation tests revealed that NheB, Hbl L2 and cytotoxicity titres of all tested *B. thuringiensis* isolates generally tended to increase together, but weakly and in a non-significant manner (Table 3A).

### 3.5. Growth Inhibition by Essential Oils (EOs)

**Figure 6:**
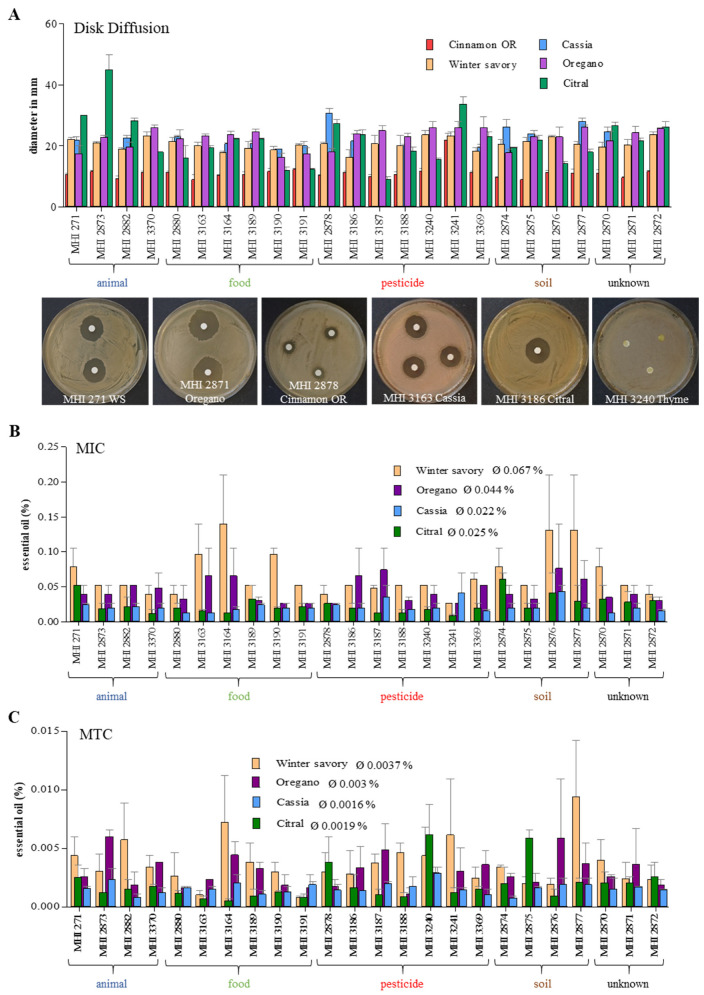
Antimicrobial activity of essential oils (EOs). (**A**) Disk diffusion assay. Approx. 10^7^ CFU/mL were plated onto CGY agar, a sterile paper disk was placed in the middle of the plate and 1 µL EO was dripped on it. Growth inhibition zones were measured after 24 h incubation at 37 °C. (**B**) The minimal inhibitory concentrations (MICs) were determined in antimicrobial broth dilution assays using 10^6^ CFU/mL. Ø: Average MIC value for all strains. (**C**) The maximal tolerated concentrations (MTCs) were determined in antimicrobial broth dilution assays using 10^3^ CFU/mL. Ø: Average MTC value for all strains. For a better overview, separate diagrams including statistics are depicted in Figures S1–S3. Blue: animal isolate, green: food, red: biopesticide, brown: soil, black: unknown origin.

Nearly all *B. thuringiensis* isolates tested in this study secreted sufficient amounts of enterotoxins under simulated intestinal conditions to harm human colon cells in a *B. cereus*-like magnitude. Thus, the antimicrobial activity of essential oils (EOs) was assessed as a preventive measure against putatively enteropathogenic *B. thuringiensis*. Initially, inhibition zones in disk diffusion assays were investigated. Except thyme, each EO was able to inhibit growth of all tested *B. thuringiensis* strains (Figure 6A and Figure S1). Susceptibility of all isolates to cinnamon OR was similar (inhibition zone approx. 11 mm), with the exception of highly sensitive MHI 3241 (22 mm). For the other EOs, especially oregano, winter savory and citral, higher strain-specific variations were detected (Figure 6A and Figure S1).

Cinnamon Chinese cassia, citral, oregano and winter savory were then chosen to evaluate MIC and MTC values by performing antimicrobial broth dilution assays (Figure 6B,C, Figures S2 and S3). Average MICs were 0.067% for winter savory, 0.044% for oregano, 0.025% for citral and 0.022% for cinnamon Chinese cassia. Average MTCs were 0.0037% for winter savory, 0.003% for oregano, 0.0019% for citral and 0.0016% for cinnamon Chinese cassia. Thus, cinnamon Chinese cassia showed the highest antimicrobial activity towards the tested *B. thuringiensis* isolates, followed by citral, oregano and winter savory. Nonetheless, the previously observed highly strain-specific antimicrobial activity was further intensified (Figure 6B,C, Figures S2 and S3). MIC and MTC values of the strain set were also statistically analysed. Results of Spearman correlation tests are summarized in Table 3B. With the exception of oregano, MIC and MTC values did not significantly correlate. MIC values of citral and cinnamon Chinese cassia (not significantly), citral and winter savory (not significantly), and winter savory and oregano (significantly) tended to increase together. This was also observed for the MTC values of cinnamon Chinese cassia and oregano, and winter savory and oregano (not significant). Thus, despite some correlation, the great variability between the tested isolates became yet again obvious. Nevertheless, soil isolate MHI 2876, which showed highest resistance to all tested EOs, as well as biopesticide isolate MHI 3241 (*B. thuringiensis* ssp. *tenebrionis*), which was most susceptible to all tested EOs except cinnamon Chinese cassia, were particularly remarkable.

## 4. Discussion

Over 400 *B. thuringiensis*-based formulations have been registered, mostly consisting of insecticidal toxins and viable spores [22]. In Germany, there are currently 16 authorized plant protection products containing *B. thuringiensis*, including biopesticides 1, 2 and 4 tested in the present study. The license for biopesticide 3 ended in 2019, its grace period ends in October 2020. *B. thuringiensis* concentrations of up to 540 g/kg are used in these products [49]. The present study aimed to investigate the enteropathogenic potential of *B. thuringiensis* as well as its susceptibility to essential oils, with special attention being paid to differences between the single isolates from soil, animals, food and commercially available biopesticides. It is already known that *B. thuringiensis*, as member of the *B. cereus* group, harbours genes encoding enterotoxins, which were also detected in the present study (see Table 1). Remarkably, all strains harboured *hbl*, which is typically found in 40–70% of *B. cereus* group isolates under investigation [26,50,51]. One older study also described a significantly higher presence of *hblCDA* in *B. thuringiensis* than in *B. cereus* [52].

Furthermore, all isolates investigated in the present study were assigned to *panC* type IV. The authors of a previous study with similar results concluded that biopesticide strains can thus indeed be present in foods [23]. Meanwhile, *B. cereus* group isolates of *panC* type IV cannot only be found in soil, water or insects, but also in conjunction with diarrhoeal outbreaks [23,27,39]. Additionally, potentially toxigenic pesticidal strains were isolated from tomatoes, cucumbers and peppers [53]. *B. thuringiensis* strains from tomatoes and bell pepper matched MLST (Multilocus Sequence Typing) types of the biopesticidal strains *B. thuringiensis* ssp. *kurstaki* ABTS-351 and *B. thuringiensis* ssp. *aizawai* ABTS-1857 [54]. In another study, isolates from food and beverage samples were indistinguishable from *B. thuringiensis* ssp. *kurstaki* from commercial biopesticides [55]. *B. thuringiensis* ssp. *kurstaki* HD1 was detected in cabbage products for human consumption [56], and a biopesticidal strain was even isolated from faecal samples of greenhouse workers who were exposed to *B. thuringiensis*-based insecticides [57].

In order to assess the potential health risk originating from (biopesticidal) *B. thuringiensis* isolates in more detail, we additionally investigated germination and motility behaviour of the strain set, which are further crucial prerequisites for the onset of the diarrhoeal disease [14,40,41,42,43,44,45,46]. Although different germination patterns emerged, spores of most isolates were able to germinate, even in cRPMI medium with preceding heat treatment mimicking re-heating of stored foods and subsequent consumption. Although highly strain-specific, no significant differences between biopesticidal and other isolates were detected, which was also true for swimming motility (see Figure 2 and Figure 3 and Table 2). A connection between motility and enterotoxin secretion in *B. thuringiensis* has already been made [58,59].

Despite all important prerequisites, production of enterotoxins in the intestine is the most relevant factor for the manifestation of the diarrhoeal disease. Although enterotoxin production by *B. thuringiensis* isolated from different sources including biopesticides has been shown in previous studies, comparative cytotoxicity assays were rarely performed [23,60,61,62,63]. Moreover, to our knowledge, the present study is the first one to compare enterotoxin production and cytotoxicity of a set of *B. thuringiensis* isolates grown under simulated intestinal conditions. Generally, enterotoxin production as well as cytotoxic activity is highly variable within enteropathogenic *B. cereus* strains. The reasons for this are still not completely explored [39,64]. A similar variability in NheB and Hbl L2 production as well as cytotoxicity towards CaCo-2 cells could be shown for the *B. thuringiensis* isolates investigated in the present study (see Figure 4 and Figure 5). Nevertheless, applying a classification scheme established for enteropathogenic *B. cereus* grown under simulated intestinal conditions [15], the majority of our *B. thuringiensis* isolates had to be classified as medium or highly toxic, including biopesticide strains (see Table 1). Hence, despite high strain-specific variations, a considerable hazardous potential due to *B. thuringiensis* isolates from various origins is indisputable.

Due to these facts, the scientific opinion of the EFSA (European Foods Safety Authority) BIOHAZ Panel (2016) is entirely comprehensible. Clear discrimination between *B. cereus* and *B. thuringiensis* in routine food and clinical diagnostics is required as well as unequivocal identification of biopesticide strains by whole genome sequencing or additional, specific markers. For an improved risk assessment, further data on dose-response and characterization of *B. thuringiensis* are urgently needed, as well as field studies after application of the biopesticides to determine pre-harvest intervals [22]. On the other hand, the major advantage of *B. thuringiensis*—its high specificity towards single insect species—must not be neglected, which led to a significant reduction of the use of chemical insecticides [1,65]. Moreover, defenders of *B. thuringiensis* claim that the genotypes of biopesticidal strains have never been found in outbreaks, and that only environmental isolates, not biopesticidal strains, have been detected in high concentrations in foodstuffs [66]. However, particularly to prove this, more data on the biopesticide strains as well as reliable tools for their clear identification are necessary.

Until these data are available, the use of EOs can be an additional preventive strategy against putatively enteropathogenic *B. thuringiensis*. It has been shown that *B. cereus* is sensitive to EOs from nutmeg, mint, clove, oregano, cinnamon, sassafras, sage, thyme and rosemary [67,68,69,70,71,72], *Satureja*, *Origanum* and *Thymus* species [73,74], lemon, orange and bergamot [75], cumin, black pepper and black cumin [76,77], *Daucus gracilis* [78], as well as carvacrol [79,80,81,82] and many others. Nevertheless, most of these studies have been conducted with only one or few representative isolates, and a comparative study including *B. cereus* strains of different origin, toxin profile, cytotoxic activity etc. is missing. Studies on the sensitivity of *B. thuringiensis* are rarely found, and—due to the high variability inside the *B. cereus* group—conclusions cannot necessarily be drawn for *B. thuringiensis*. Only two earlier studies showed susceptibility of *B. thuringiensis* towards EOs of *Thymus vulgaris* [83] and *Ferulago angulata* [84]. In contrast to that, none of the tested strains in the present study was inhibited by thyme oil (Figure 6), which once more underlines the high variability of EO effects. Comparing average MIC values, we determined an antimicrobial activity of cinnamon Chinese cassia > citral > oregano > winter savory, which largely corresponds with earlier studies on *B. cereus* [72]. However, as in all tests applied in this study, strain-specific differences must be considered.

## 5. Conclusions

Altogether, the present study stresses the enteropathogenic potential of *B. thuringiensis*, even of biopesticidal strains, which were generally indistinguishable from other isolates in the applied tests. Application of these pesticides might thus indeed constitute a hazard to human health. On the other hand, the use of *B. thuringiensis* in agriculture significantly reduced the application of chemical insecticides. We conclude that banning of *B. thuringiensis* biopesticides is not implementable due to its various advantages. Nevertheless, the applied strains have to be extremely well characterized (for instance by whole genome sequencing) and labelled to provide gapless information about their safety and to eliminate any human health hazard. At this, routine discrimination between *B. cereus* and *B. thuringiensis* is as important as controlled application of the biopesticides. Additionally, clear labelling of foods treated with *B. thuringiensis* can benefit consumers.

Until then, the use of essential oils as antimicrobials might diminish the risks by reducing the level of contamination, especially in times of increased antibiotic resistance and enhanced consumers’ demand for natural antimicrobial agents. It can also be concluded from the present study that, whenever predicting the hazardous potential or using antimicrobials, the high variety within *B. cereus* group strains, even *B. thuringiensis*, must be taken into account, whether it be their ability to produce enterotoxins, their cytotoxic activity, or their resilience towards antimicrobials.

## Figures and Tables

**Table 1:** Overview of the 24 *B. thuringiensis* strains used in this study. Origin of the isolates: A: animal, F: food, P: pesticide, S: soil, U: undefined. +: positive PCR result for toxin gene. −: negative PCR result for toxin gene. *: NheB production determined in sandwich enzyme immunoassays (EIAs) after growth under simulated intestinal conditions according to Jessberger et al. [15]. **: Cytotoxicity towards CaCo-2 cells after growth under simulated intestinal conditions according to Jessberger et al. [15].

Internal Number (MHI)	Other Designation	Source	Origin	*panC* Type	*cry*	*nhe*	*hbl*	*ces*	*cytK1*	*cytK2*	NheB *	Toxicity **
271	*B. thuringiensis* Berliner 1915, DSM 6029	Animal tissue	A	IV	1	+	+	−	−	+	High	High
2873	*B. thuringiensis* Berliner 1915, ATCC^®^ 10792™, DSM 2046	Mediterranean flour moth	A	IV	1	+	+	−	−	+	High	High
2882	*B. thuringiensis* ssp. *israelensis*, BGSC 4Q2 (HD500)	Culicidae larva	A	IV	4	+	+	−	−	+	High	Medium
3370	*B. thuringiensis* Bt 407	*Ephestia kuehniella* larva; Cry-	A	IV	−	+	+	−	−	+	High	High
2880	*B. thuringiensis* WSBC 28025	Vegetables for infant food	F	IV	1, 2	+	+	−	−	+	Medium	High
3163	*B. thuringiensis*	Ready-to-eat salad 1	F	IV	1, 2	+	+	−	−	+	High	High
3164	*B. thuringiensis*	Ready-to-eat salad 2	F	IV	1, 2	+	+	−	−	+	Medium	High
3189	*B. thuringiensis*	Ready-to-eat salad 3	F	IV	1, 2	+	+	−	−	+	Low	Medium
3190	*B. thuringensis*	Ready-to-eat salad 4	F	IV	1, 2	+	+	−	−	+	Low	Medium
3191	*B. thuringiensis*	Ready-to-eat salad 5	F	IV	1	+	+	−	−	+	Low	High
2878	*B. thuringiensis* ssp. *tenebrionis*	Undefined biopesticide	P	IV	3	+	+	−	−	−	Low	Medium
3186	*B. thuringiensis* ssp. *aizawai*	Biopesticide granulate 1	P	IV	1, 2	+	+	−	−	+	Low	High
3187	*B. thuringiensis* ssp. *aizawai*	Biopesticide granulate 1	P	IV	1, 2	+	+	−	−	+	Low	Medium
3188	*B. thuringensis*	Isolate from Biopesticide 1	P	IV	1, 2	+	+	−	−	+	Low	High
3240	*B. thuringensis* ssp. *kurstaki*	Biopesticide 2	P	IV	1, 2	+	+	−	−	+	Medium	High
3241	*B. thuringiensis* ssp. *tenebrionis*	Biopesticide 3, solids	P	IV	3	+	+	−	−	−	Medium	Low
3369	*B. thuringiensis* ssp. *aizawai*	Biopesticide 4	P	IV	1, 2	+	+	−	−	+	Low	High
2874	*B. thuringiensis* ssp. *kurstaki*, PO1	Soil	S	IV	1	+	+	−	−	+	High	Medium
2875	*B. thuringiensis* ssp. *kurstaki*, PO6	Soil	S	IV	1	+	+	−	−	+	High	High
2876	*B. thuringiensis* ssp. *kurstaki*, PO10	Soil	S	IV	1	+	+	−	−	+	Medium	High
2877	*B. thuringiensis* ssp. *kurstaki*, PO14	Soil	S	IV	1	+	+	−	−	+	High	High
2870	*B. thuringiensis* ssp. *entomocidus* bv. *subtoxicus*, IEBC-T06A001	Canada	U	IV	1, 2	+	+	−	−	+	Medium	Low
2871	*B. thuringiensis*	Undefined	U	IV	1, 2	+	+	−	−	+	Medium	Medium
2872	*B. thuringiensis* HER 1404	Undefined	U	IV	1, 2	+	+	−	−	+	Medium	Medium

**Table 2:** Germination of *B. thuringiensis* spores. Germination was measured as the decrease of OD_620_ per time. OD_620_ at time point 0 was set to 100%. To compare germination rates of the 24 tested isolates, OD_620_ (in %) after 15 min incubation is shown. Shown are means and standard deviations of at least two biological replicates, each comprising three technical replicates. Origin of the isolates: A: animal, F: food, P: pesticide, S: soil, U: unknown. Strains are sorted according to their germination patterns: A. Minimal to no germination. B. Strong germination in nutrient-rich medium plus heat activation with little germination in cRPMI medium. C. Heat- and nutrient-dependent germination. D. Heat- and nutrient-independent germination. E. Germination heat-dependent, but mostly nutrient-independent.

Internal Number (MHI)	Origin	Germination (OD_620_ in %) after 15 min				Germination Pattern
		CGY	CGY 80 °C	cRPMI	cRPMI 80 °C	
2870	U	94.2 ± 0.6	96.4 ± 0.5	97.8 ± 1.3	98.1 ± 0.6	A
2873	A	96.6 ± 0.8	96.8 ± 0.1	98.5 ± 1.3	94.2 ± 1.9	A
2880	F	93.5 ± 2.8	71.4 ± 1.4	98.1 ± 1.5	98.9 ± 1.4	B
2882	A	85.2 ± 4.1	78.0 ± 1.0	97.2 ± 1.6	96.4 ± 2.8	B
3163	F	94.8 ± 0.6	86.5 ± 1.2	98.8 ± 0.7	97.6 ± 1.3	B
3164	F	94.1 ± 0.3	86.9 ± 2.5	98.6 ± 1.7	96.6 ± 1.1	B
3186	P	98.5 ± 0.3	88.5 ± 2.5	99.8 ± 0.3	100.1 ± 0.6	B
3187	P	98.9 ± 0.9	90.4 ± 2.7	99.4 ± 0.7	99.7 ± 0.5	B
3188	P	96.0 ± 0.4	78.8 ± 0.3	98.4 ± 0.5	100.5 ± 1.1	B
3189	F	96.2 1± 1.6	88.2 ± 2.3	98.6 ± 0.4	100.6 ± 0.8	B
3190	F	98.4 ± 0.6	94.8 ± 2.0	99.1 ±0.9	99.6 ± 0.2	B
3191	F	93.1 ± 0.4	77.2 ± 0.7	99.3 ± 0.8	99.2 ± 0.1	B
2871	U	90.6 ± 3.2	82.4 ± 2.1	89.7 ± 4.4	91.3 ± 3.0	C
2872	U	81.5 ± 1.2	72.6 ± 2.9	96.5 ± 0.2	93.4 ± 0.4	C
3240	P	98.1 ± 1.9	79.2 ± 1.3	90.3 ± 5.8	93.0 ± 1.9	C
3241	P	86.5 ± 7.1	75.6 ± 10.9	119.1 ± 24.7	85.2 ± 11.1	C
3369	P	87.3 ± 1.6	75.2 ± 2.7	96.9 ± 1.9	95.0 ± 1.6	C
2874	S	82.9 ± 31.5	62.0 ± 4.1	74.4 ± 1.5	81.7 ± 1.5	D
2875	S	78.8 ± 6.2	74.8 ± 1.9	83.9 ± 2.0	82.7 ± 1.8	D
2876	S	87.1 ± 1.9	89.9 ± 1.0	90.1 ± 4.9	89.1 ± 0.4	D
2877	S	81.3 ± 7.8	75.6 ± 9.6	91.3 ± 2.2	93.5 ± 1.4	D
3370	A	71.9 ± 6.8	64.5 ± 8.2	78.6 ± 9.1	78.2 ± 9.5	D
271	A	95.2 ± 1.3	81.9 ± 3.3	97.7 ± 0.6	80.2 ± 0.1	E
2878	P	96.1 ± 1.8	88.8 ± 2.2	96.4 ± 2.0	92.3 ± 2.1	E

**Table 3:** Results of Spearman correlation tests. Correlation between reciprocal titres from EIAs and cytotoxicity tests (**A**), as well as correlation between MICs and MTCs of EOs (**B**) was determined for the 24 tested *B. thuringiensis* isolates. Pairs with positive correlation coefficients are shown, which tended to increase together. R: correlation coefficient. *: significant correlation (*p*-value ≤ 0.05). ns: not significant.

**A**	WST-1 CGY		NheB CGY
NheB CGY Hbl L2 CGY	R = 0.37 (ns) R = 0.237 (ns)	NheB cRPMI Hbl L2 CGY	R = 0.35 (ns) R = 0.297 (ns)
			
	WST-1 cRPMI		NheB cRPMI
NheB cRPMI	R = 0.381 (ns)	Hbl L2 cRPMI	R = 0.283 (ns)
Hbl L2 cRPMI	R = 0.375 (ns)		
			
	WST-1 CGY		Hbl L2 CGY
WST-1 cRPMI	R = 0.237 (ns)	Hbl L2 cRPMI	R = 0.13 (ns)
			
**B**	MIC Citral		MIC Oregano
MTC Citral	R = 0.35 (ns)	MTC Oregano	R = 0.65 (*)
			
	MIC Cassia		MIC Winter s.
MIC Citral	R = 0.126 (ns)	MIC Citral	R = 0.322 (ns)
		MIC Oregano	R = 0.446 (*)
			
	MTC Cassia		MTC Winter s.
MTC Oregano	R = 0.285 (ns)	MTC Oregano	R = 0.16 (ns)

## Supplementary Materials

The following are available online at https://www.mdpi.com/2304-8158/9/10/1484/s1, Table S1: Primers used in this study, Figure S1: Disk diffusion assay, Figure S2: Determination of minimal inhibitory concentrations (MICs) in antimicrobial broth dilution assays, Figure S3: Determination of maximal tolerated concentrations (MTCs) in antimicrobial broth dilution assays.
