# Supplementary material for: Enteropathogenic Potential of Bacillus thuringiensis Isolates from Soil, Animals, Food and Biopesticides
Source: Foods. 2020 Oct 17;9(10):1484. doi: 10.3390/foods9101484 (PMC7603059; doi:10.3390/foods9101484)
Supplement: Supplementary file 1 [file foods-09-01484-s001.zip › Fig. S1.pdf]

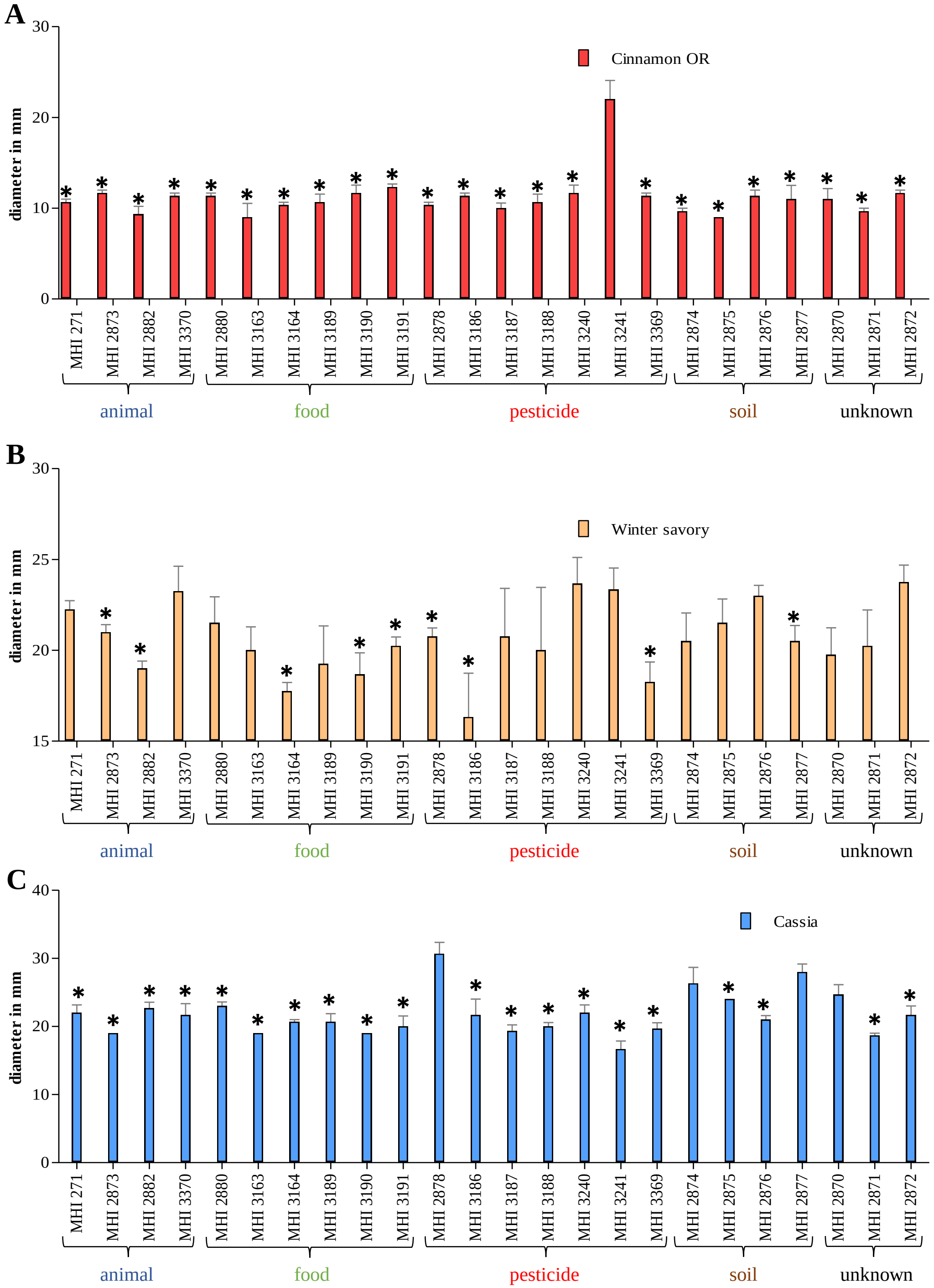

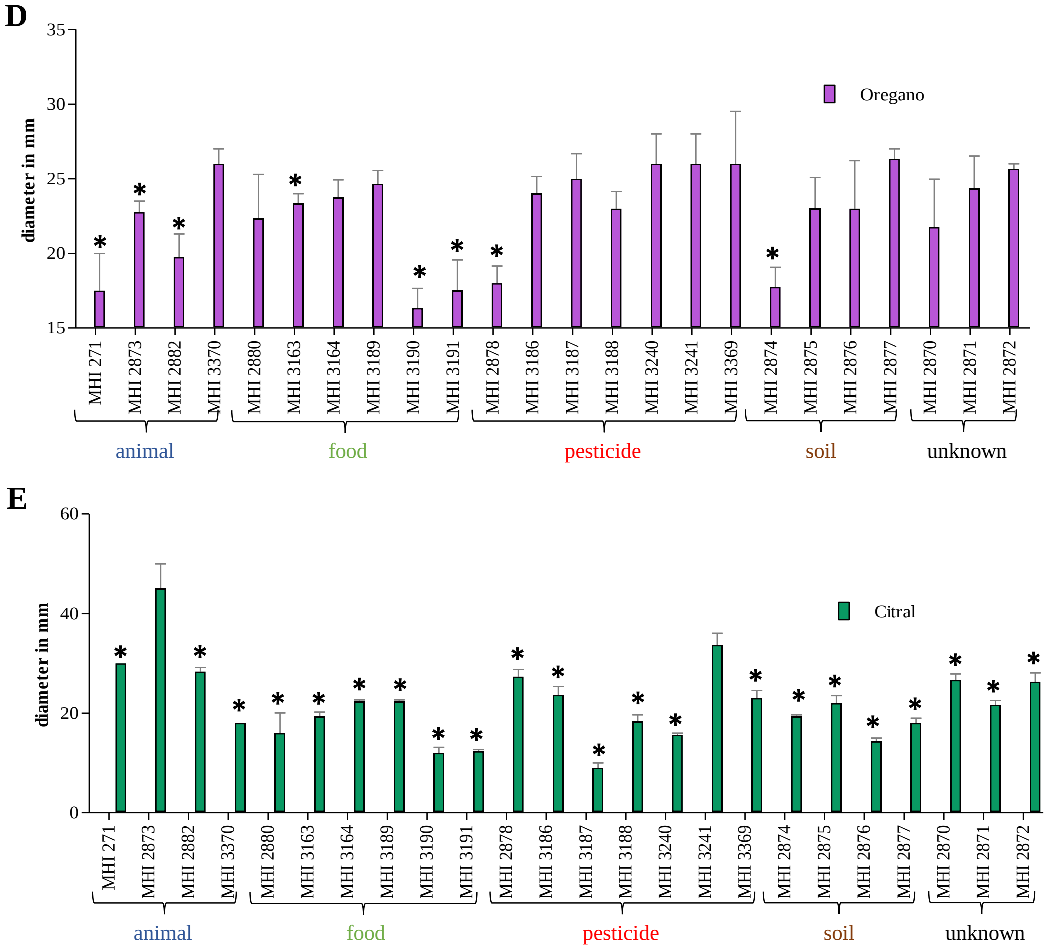

Fig. S1: Disk Diffusion assay. Approx. 10^7 CFU/ml were plated onto CGY agar, a sterile paper disk was placed in the middle of the plate and 1 µl EO was dripped on it. Growth inhibition zones were measured after 24 hours incubation at 37 °C. For an improved overview, each EO is depicted in a separate diagram. **A.** Cinnamon OR. **B.** Winter savory. **C.** Cinnamon Chinese cassia. **D.** Oregano. **E.** Citral. *: Significant difference compared to the isolate with highest diameter (p value < 0.05; unpaired t-test, 95% confidence interval). Blue: animal isolate, green: food, red: biopesticide, brown: soil, black: unknown origin.
