# Supplementary material for: Enteropathogenic Potential of Bacillus thuringiensis Isolates from Soil, Animals, Food and Biopesticides
Source: Foods. 2020 Oct 17;9(10):1484. doi: 10.3390/foods9101484 (PMC7603059; doi:10.3390/foods9101484)
Supplement: Supplementary file 1 [file foods-09-01484-s001.zip › Fig. S3.pdf]

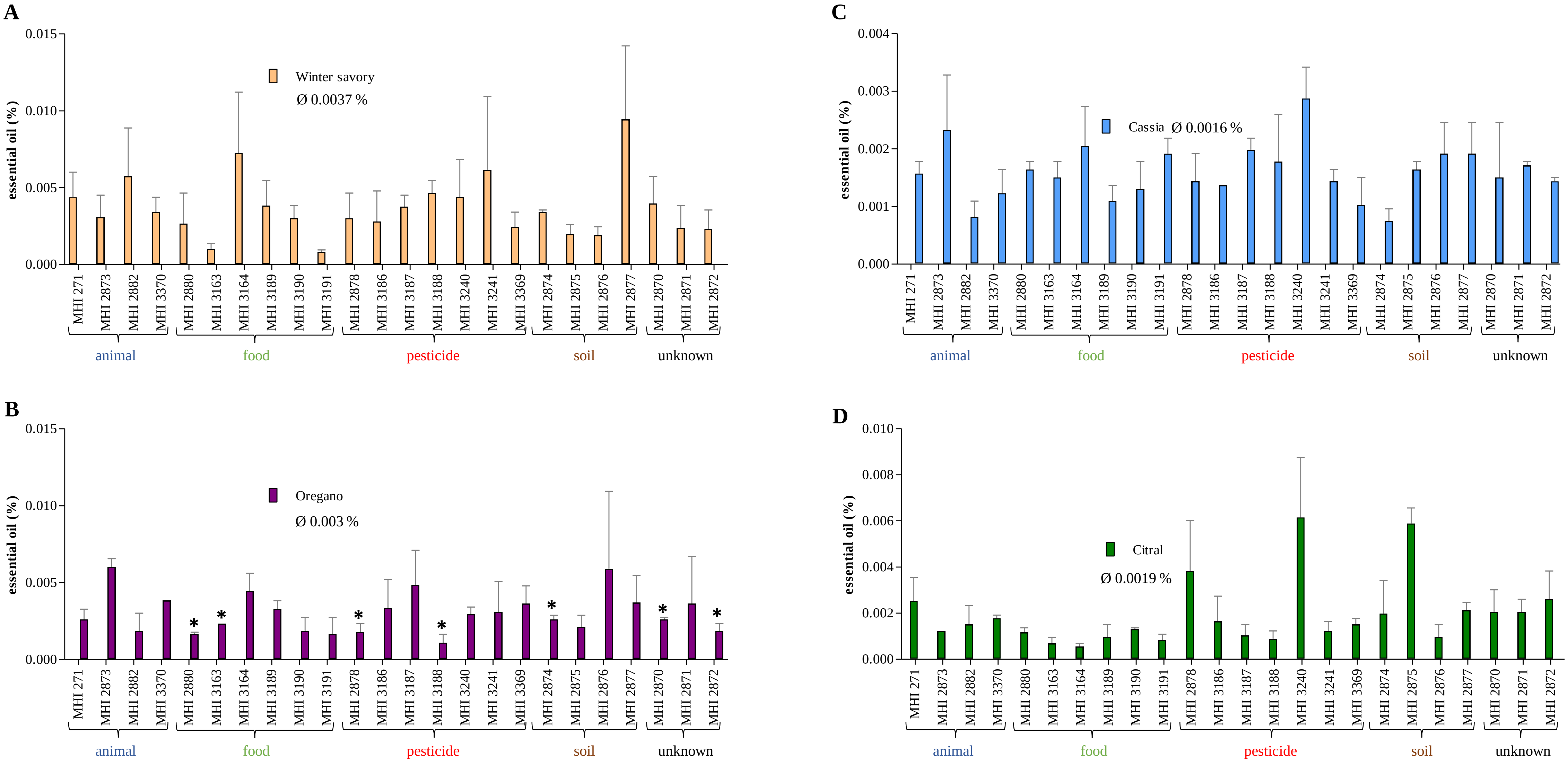

Fig. S3: Determination of maximal tolerated concentrations (MTCs) in antimicrobial broth dilution assays. For an improved overview, each EO is depicted in a separate diagram. **A.** Winter savory. **B.** Oregano. **C.** Cinnamon Chinese cassia. **D.** Citral. *: Significant difference compared to the isolate with highest MTC (p value < 0.05; unpaired t-test, 95% confidence interval). Blue: animal isolate, green: food, red: biopesticide, brown: soil, black: unknown origin.
