# Supplementary material for: Enteropathogenic Potential of Bacillus thuringiensis Isolates from Soil, Animals, Food and Biopesticides
Source: Foods. 2020 Oct 17;9(10):1484. doi: 10.3390/foods9101484 (PMC7603059; doi:10.3390/foods9101484)
Supplement: Supplementary file 1 [file foods-09-01484-s001.zip › Table S1.docx]

**Table S1: Primers used in this study.**

| **Primer** | **Sequence 5’ – 3’** | **Gene** | **Ta**  **(°C)** | **Product**  **(bp)** | **Reference** |
| --- | --- | --- | --- | --- | --- |
| panC-fw  panC-rev | ATGAAAATCGTAACTACAGTGC  TTATTTAACCGTTAATGTTATATTGTC | *panC* | 50 | 849 | - |
| cry1-un-fw  cry1-un-rev | CATGATTCATGCGGCAGATAAAC  TTGTGACA CTTCTGCTTCCCATT | *cry1* | 53 | 276 | [24] |
| cry2-un-fw  cry2-un-rev | GTTATTCTTAATGCAGATGAATGGG  CGGATAAAATAATCTGGGAAATAGT | *cry2* | 53 | 700 | [24] |
| cry3-un-fw  cry3-un-rev | CGTTATCGCAGAGAGATGACATTAAC  CATCTGTTGTTTCTGGAGGCAAT | *cry3* | 55 | 600 | [24] |
| cry4-un-fw  cry4-un-rev | GCATATGATGTAGCGAAACAAGCC  GCGTGACATACCCATTTCCAGGTCC | *cry4* | 55 | 439 | [24] |
| cry7/8-un-fw  cry7/8-un-rev | AAGCAGTGAATGCCTTGTTTAC  CTTCTAAACCTT GACTACTT | *cry7/8* | 50 | 420 | [24] |
| 45c1  45c2 | GAG GGG CAA ACA GAA GTG AA  TGC GAA CTT TTG ATG ATT CG | *nheA* | 49 | 186 | [25] |
| L2aF  L2aR | CGA AAA TTA GGT GCG CAA TC  TAA TAT GCC TTG CGC AGT TG | *hblC* | 49 | 411 | [25] |
| CesF1  CesR2 | GGT GAC ACA TTA TCA TAT AAG GTG  GTA AGC GAA CCT GTC TGT AAC AACA | *ces* | 49 | 1271 | [25] |
| F2  R7 | AAC AGA TAT CGG TCA AAA TGC  CGT GCA TCT GTT TCA TGA GG | *cytK1* | 49 | 623 | [25] |
| CK-F2  CK-R5 | ACA GAT ATC GGT CAA AAT GC  CAA GTT ACT TGA CCT GTT GC | *cytK2* | 50 | 421 | [26] |
|  |  |  |  |  |  |
|  |  |  |  |  |  |
|  |  |  |  |  |  |
